# Identification and characterization of lysophosphatidylcholine 14:0 as a biomarker for drug-induced lung disease

**DOI:** 10.1038/s41598-022-24406-z

**Published:** 2022-11-17

**Authors:** Kosuke Saito, Akihiko Gemma, Koichiro Tatsumi, Noboru Hattori, Atsuhito Ushiki, Kenji Tsushima, Yoshinobu Saito, Mitsuhiro Abe, Yasushi Horimasu, Takeru Kashiwada, Kazuhiko Mori, Motonobu Sato, Takayoshi Nishiya, Kazuhiko Takamatsu, Yuchen Sun, Noriaki Arakawa, Takashi Izumi, Yasuo Ohno, Yoshiro Saito, Masayuki Hanaoka

**Affiliations:** 1 Division of Medical Safety Science, National Institute of Health Sciences, Kawasaki, 210-9501 Japan; 2 Department of Pulmonary Medicine and Oncology, Graduate School of Medicine, Nippon Medical School, Tokyo, 113-8603 Japan; 3 Department of Respirology (B2), Graduate School of Medicine, Chiba University, Chiba, 260-8677 Japan; 4 Department of Respiratory Medicine, Hiroshima University Hospital, Hiroshima, 734-8551 Japan; 5 First Department of Internal Medicine, Shinshu University School of Medicine, Matsumoto, 390-8621 Japan; 6 School of Medicine, International University of Health and Welfare, Narita, 286-8686 Japan; 7 Daiichi Sankyo RD Novare Co., Ltd., Tokyo, 134-8630 Japan; 8 Astellas Pharma Inc., Tsukuba, 305-8585 Japan; 9 Kihara Memorial Foundation, Yokohama, 230-0045 Japan

**Keywords:** Biomarkers, Biomarkers, Translational research

## Abstract

Drug-induced interstitial lung disease (DILD) occurs when drug exposure causes inflammation of the lung interstitium. DILD can be caused by different types of drugs, and some DILD patterns results in a high mortality rate; hence, DILD poses a serious problem in clinical practice as well as drug development, and strategies to diagnose and distinguish DILD from other lung diseases are necessary. We aimed to identify novel biomarkers for DILD by performing lipidomics analysis on plasma samples from patients with acute and recovery phase DILD. Having identified lysophosphatidylcholines (LPCs) as candidate biomarkers for DILD, we determined their concentrations using validated liquid chromatography/mass spectrometry biomarker assays. In addition, we evaluated the ability of LPCs to discriminate patients with acute phase DILD from those with recovery phase DILD, DILD-tolerant, or other lung diseases, and characterized their association with clinical characteristics. Lipidomics analysis revealed a clear decrease in LPC concentrations in the plasma of patients with acute phase DILD. In particular, LPC(14:0) had the highest discriminative index against recovery phase and DILD-tolerant patients. LPC(14:0) displayed no clear association with causal drugs, or subjects’ backgrounds, but was associated with disease severity. Furthermore, LPC(14:0) was able to discriminate between patients with DILD and other lung diseases, including idiopathic interstitial pneumonia and lung disease associated with connective tissue disease. LPC(14:0) is a promising biomarker for DILD that could improve the diagnosis of DILD and help to differentiate DILD from other lung diseases, such as idiopathic interstitial pneumonia and connective tissue disease.

## Introduction

Drug-induced interstitial lung disease (DILD) is a group of diffuse parenchymal lung disorders. DILD is caused by inflammation of the lung interstitium following exposure to over 380 known drugs, including cancer chemotherapy agents (e.g., paclitaxel and gemcitabine), amiodarone, and monoclonal antibody therapies (e.g., nivolumab and pembrolizumab)^1–4^. Although DILD exhibits numerous patterns in histological diagnosis, typically, diffuse alveolar damage (DAD), organizing pneumonia (OP), non-specific interstitial pneumonia (NSIP), eosinophilic pneumonia, and hypersensitivity pneumonia are observed^5,6^. These histopathological patterns as well as clinical phenotypes and computed tomography (CT) images of DILD vary significantly, even between patients receiving the same drug. As DILD is a serious adverse drug reaction that poses problems in drug development and clinical practice, new approaches are urgently required to specifically diagnose DILD. In addition, the variability of DILD properties makes distinguishing DILD from other related lung diseases complicated, including interstitial pneumonias caused by other factors^4–7^. Thus, along with DILD diagnosis, approaches to distinguish DILD from other lung diseases, especially idiopathic interstitial pneumonia (IIP), are also required.

DILD is traditionally diagnosed through comprehensive clinical evaluations, including laboratory tests for basic blood parameters and known lung biomarkers, chest radiography and/or high-resolution CT (HRCT), pulmonary function testing, and, if necessary, invasive procedures such as bronchoscopy^4–8^. Since biomarker tests have particular advantages in terms of low cost, minimal invasiveness, and ease of handling by general physicians, they are playing an increasingly important role in clinical drug use and development. The known lung biomarkers used to assist the diagnosis of DILD are surface protein-D (SP-D) and Krebs von den Lungen-6 (KL-6), which are glycoproteins produced by type II pneumocytes^9–11^. A prospective study revealed that KL-6 levels are increased in 53% of patients with DILD, which correlates with DAD and extensive lung involvement^12^. Meanwhile, it has been reported that SP-D can be used to discriminate between everolimus-treated patients with (*n* = 12) and without (*n* = 13) DILD^13^. However, these known biomarkers have also been shown to be changed by other lung diseases, such as lung cancer (LuCa), IIP, and lung disease associated with connective tissue disease (CTD)^12,14–16^. Since DILD can be caused by a broad spectrum of drugs and the underlying lung diseases vary between DILD patients, more specific biomarkers are required to facilitate the diagnosis of DILD and select appropriate treatments in a timely manner.

Technical advances in omics approaches have allowed the comprehensive analysis of circulating molecules, including RNA, proteins, and metabolites, to improve the discovery of candidate biomarkers. Lipidomics is a relatively recent omics approach that targets lipids^17–19^, a major class of metabolites that constitute the structural components of cells, organelles, and vesicle membranes, and act as a source of energy and/or cell signaling molecules. Accordingly, lipids are considered suitable targets for biomarker development. In this study, we used lipidomics analysis to obtain novel biomarkers for DILD, from plasma samples obtained from patients with acute and recovery phase DILD. Having identified lysophosphatidylcholines (LPCs) as candidate biomarkers for DILD, we validated these biomarkers in DILD-tolerant patients prescribed with DILD-causing drugs for at least 12 weeks. In addition, we verified the association between LPC(14:0), the most promising biomarker candidate for DILD, and the clinical characteristics of patients with DILD, as well as their ability to discriminate between DILD and other lung diseases.

## Materials and methods

### Subjects

All patients were recruited from Shinshu University, Nippon Medical School, Chiba University, and Hiroshima University. Healthy volunteers were recruited from the Yaesu Sakura-Dori Clinic. DILD was diagnosed by respiratory specialists according to the following Japanese diagnostic criteria^6^ that ameliorated the diagnostic criteria reported by Camus et al.^5^: 1) History of ingestion of a drug known to induce lung injury; 2) the clinical manifestations have been reported to be induced by a drug; 3) other causes of the clinical manifestations could be ruled out; and 4) improvement of the clinical manifestations after drug discontinuation.

Based on HRCT findings, DILD was classified into four patterns: DAD, NSIP, OP, and others, by respiratory specialists at each hospital. Some patients displayed multiple patterns.

DILD recovery was determined at least two weeks after the onset of DILD by respiratory specialists at each hospital, based on the improvement of clinical symptoms, lung imaging findings (e.g., HRCT), and oxygenation status (e.g., SpO_2_). Patients who were prescribed DILD-causing drugs without DILD for at least 12 weeks were enrolled in the DILD-tolerant group. Differences in general and background diseases and clinical characteristics among DILD groups were evaluated using Student’s *t*-test for numerical factors and the chi-square test for categorical factors. Patients with LuCa, bacterial pneumonia (BaPn), nontuberculous mycobacteriosis (NoMy), IIP, CTD, chronic obstructive pulmonary disease (COPD), or bronchial asthma (BrAs) were also diagnosed by respiratory specialists.

### Sample collection

Blood samples were collected into vacuum blood collection tubes containing 7 mL EDTA-2Na by venipuncture (Venoject II, Terumo, Tokyo, Japan) and were immediately centrifuged at 2500 × *g* and 4 °C for 10 min. Plasma was dispensed into screw-capped polypropylene tubes and stored at − 80 °C within 2–4 h of drawing blood.

### Lipidomics and candidate biomarker discovery

Lipidomics was performed as described previously^20^. The processed lipid level data are presented in Supplementary information 2. Lipids confirmed by fragment ions were used for data analysis. Significant differences in lipid levels were assessed using the false discovery rate (FDR)-adjusted Welch’s *t*-test, and candidate biomarkers were identified using the effect size (Hedge’s g).

### LPC validated liquid chromatography/mass spectrometry (LC/MS) biomarker assay and biomarker validation and characterization

A validated LC/MS biomarker assay was performed on the LPCs as described previously^21^, with some modifications. The modified details and validated assay parameters and performance are described in Supplementary information 1 and Supplementary information 3. The determined LPC concentrations and associated clinical characteristics of patients with and without DILD are presented in Supplementary information 4.

### Statistical analysis

LPC concentrations were compared between two or more groups using Student’s *t*-tests or ANOVA with Tukey’s post-hoc test in GraphPad Prism 9 (GraphPad Software, San Diego, CA, USA). The discrimination ability of different LPCs was assessed using the area under the curve (AUC) of receiver operating characteristic (ROC) analysis using GraphPad Prism 9. The correlation between LPC concentration and severity of symptoms in DILD was calculated using Pearson’s correlation coefficient. Multivariable logistic regression analysis of LPC concentration and associated clinical characteristics was performed using R 3.6.0 software (R Foundation for Statistical Computing, Vienna, Austria).

### Ethics approval

This study was conducted in accordance with the Declaration of Helsinki and was approved by the Ethics Committee of the National Institute of Health Science (NIHS) (257 and 259 for NIHS; 261 and 263 for Kihara Memorial Foundation), Shinshu University (3318 and 4716), Nippon Medical School (27-11-514), Chiba University (2265), Hiroshima University (E-245), Daiichi Sankyo Co., Ltd. (15-0504-00), and Astellas Pharma Inc. (000043).

### Consent to participate

Written informed consent was obtained from all study participants.

## Results

### Lipidomics screening of candidate biomarkers for DILD

**Figure 1:**
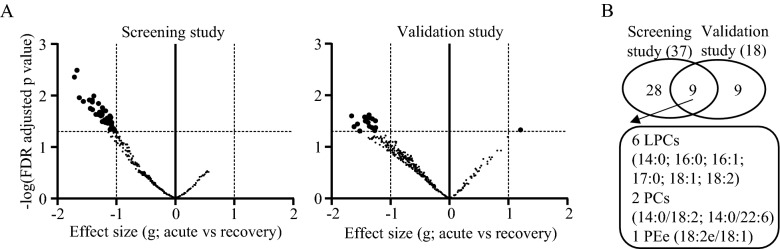
Lipidomics screening of candidate DILD biomarkers. (**A**) Individual lipid plot of false discovery rate (FDR) and effect size (g) from lipidomics analyses comparing acute and recovery phase DILD patients. (**B**) Venn diagram of significantly different lipids between acute and recovery phase DILD patients from the screening and validation cohorts.

First, we screened candidate biomarkers in 43 patients with DILD (43 acute phase samples; 30 recovery phase samples), whose clinical characteristics are summarized in Supplementary information 5. The screening set included 21 patients (21 acute phase; 17 recovery phase), while the validation set included 22 patients (22 acute phase; 13 recovery phase). Lipidomic analysis of the plasma samples identified 396 lipids (Supplementary information 2) that were quantified as arbitrary lipid units normalized to an internal standard (phosphatidylcholine(12:0/12:0)). As seen in Fig. 1A, a number of lipids exceeded the stringent threshold (FDR-adjusted adjusted *p *< 0.05 and effect size (|g|) > 1) level of difference between the acute and recovery phase samples, including 37 and 18 in the screening and validation sets, respectively (Fig. 1B). Then, lipids exceeding the threshold in both the screening and validation studies were extracted, which resulted in nine lipids being identified as candidate biomarkers (Fig. 1B), all of which were expressed at lower levels in the acute phase samples compared to that in the recovery phase samples. Since six of the nine candidate biomarkers were LPCs, we focused on LPCs in subsequent experiments.

### Validation of LPCs as DILD biomarkers

To validate LPCs as candidate biomarkers for DILD, we established and validated the LC/MS biomarker assay to determine LPC plasma concentrations. Due to the availability of standards and the larger effect size of saturated/monounsaturated LPCs, we targeted the following eight LPCs: LPC(14:0), LPC(15:0), LPC(16:0), LPC(17:0), LPC(18:0), LPC(18:1), LPC(19:0), and LPC(20:0), based on the availability of analytical standards. The validated assay parameters (Supplementary information 3) were compatible with the acceptance criteria for bioanalytical drug validation methods (as described in Supplementary information 1). A total of 102 patients with DILD (102 acute phase; 59 recovery phase) and 31 DILD-tolerant patients were recruited for the validation assay, and their clinical characteristics are summarized in Table 1 and listed individually in Supplementary information 4.

**Table 1:** Summary of clinical characteristics of DILD and DILD-tolerant patients used for LPC biomarker assay.

	Factor	Category	DILD acute	DILD recovery		DILD-tolerant	
General	total no. of subjects		102	59		31	
General	(paired subjects)		59	59	stat^#^		stat^$^
General	Age	Range/median (no. of sub.)	32–86/69 (100)	32–85/69 (58)	ns	33–83/69 (31)	ns
General	Gender	Female/male (no. of sub.)	38/64 (102)	24/35 (59)	ns	13/18 (31)	ns
General	BMI	Range/median (no. of sub.)	14.0–28.7/21.3 (100)	14.6–28.7/22.6 (58)	ns	16.3–38.1/21.6 (31)	ns
General	Smoking habit	No/Yes (no. of .sub.)	34/63 (97)	19/38 (57)	ns	10/21 (31)	ns
General	Chest radiation	No/Yes (no. of sub.)	90/11 (101)	53/6 (59)	ns	27/4 (31)	ns
Underlying diseases	Lung cancer	No/Yes (no. of sub.)	72/30 (102)	49/10 (59)	ns	2/29 (31)	*p *< 0.0001
Underlying diseases	Other cancer	No/Yes (no. of sub.)	54/48 (102)	29/30 (59)	ns	28/3 (31)	*p *= 0.0002
Underlying diseases	High blood pressure	No/Yes (no. of sub.)	77/25 (102)	45/14 (59)	ns	19/12 (31)	ns
Underlying diseases	Diabetes	No/Yes (no. of sub.)	83/19 (102)	46/13 (59)	ns	26/5 (31)	ns
Underlying diseases	Lipid disorder	No/Yes (no. of sub.)	83/19 (102)	49/10 (59)	ns	27/4 (31)	ns
Underlying diseases	Heart disease	No/Yes (no. of sub.)	81/21 (102)	46/13 (59)	ns	29/2 (31)	ns
Clinical tests	WBC (cells/μl)	Range/median (no. of sub.)	2200–105100/7040 (102)	1450–21210/6215 (58)		2910–9760/5960 (31)	
Clinical tests	SP-A (ng/ml)	Range/median (no. of sub.)	16–257/72.3 (57)	17.6–118.2/46.7 (30)		21.6–145.9/40.6 (13)	
Clinical tests	SP-D (ng/ml)	Range/median (no. of sub.)	17.2–1720/205 (76)	8.6–401/108 (33)		21–227/88.2 (14)	
Clinical tests	KL-6 (U/ml)	Range/median (no. of sub.)	133.4–5366/887 (101)	106–4256/518 (47)		170–2744/322 (21)	
Clinical tests	CRP (mg/dl)	Range/median (no. of sub.)	0.05–30.97/4.49 (102)	0.01–19.3/0.3 (55)		0.01–16.6/0.17 (30)	
Clinical tests	LDH (U/l)	Range/median (no. of sub.)	119–812/276 (101)	146–481/220.5 (56)		25–442/217 (31)	
DILD pattern	DAD	No/Yes (no. of sub.)	79/21 (100)	NA		NA	
DILD pattern	OP	No/Yes (no. of sub.)	58/42 (100)	NA		NA	
DILD pattern	NSIP	No/Yes (no. of sub.)	64/36 (100)	NA		NA	
DILD pattern	Other	No/Yes (no. of sub.)	86/14 (100)	NA		NA	
Causal drug categories	L01BC; Pyrimidine analogs	No/Yes (no. of sub.)	87/15 (102)	NA		30/1 (31)*	
Causal drug categories	L01CD; Taxanes	No/Yes (no. of sub.)	80/22 (102)	NA		19/12 (31)*	
Causal drug categories	L01XC; Monoclonal antibodies	No/Yes (no. of sub.)	82/20 (102)	NA		26/5 (31)*	
Causal drug categories	L01XE; Protein kinase inhibitors	No/Yes (no. of sub.)	85/17 (102)	NA		15/16 (31)*	
Symptom severities of DILD	Cough (grade)	Range/median (no. of sub.)	0–3/1 (96)	NA		NA	
Symptom severities of DILD	Breathlessness (grade)	Range/median (no. of sub.)	0–3/1 (96)	NA		NA	
Symptom severities of DILD	Body temperature	Range/median (no. of sub.)	35–39/36.7 (98)	NA		NA	
Symptom severities of DILD	Oxygen administration	No/Yes (no. of sub.)	78/22 (100)	NA		NA	
Symptom severities of DILD	SpO_2_	Range/median (no. of sub.)	50–99/95.5 (78)	NA		NA	

*Prescribed DILD-causing drugs without DILD for at least 12 weeks, stat#: Statistical comparison between DILD acute and DILD recovery, stat$: Statistical comparison between DILD acute and DILD-tolerant.

**Figure 2:**
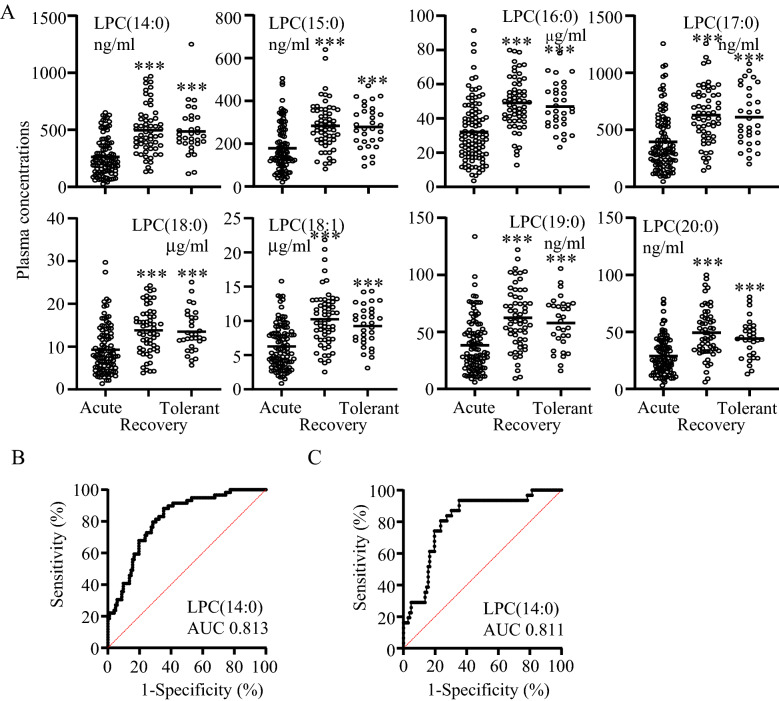
Validation of LPCs as DILD biomarkers. (**A**) Individual plot of plasma LPC concentrations in acute and recovery phase DILD and DILD-tolerant patients. ***p < 0.001 *vs.* acute phase. (**B**) ROC curve of LPC(14:0) concentrations between acute and recovery phase DILD patients. (**C**) ROC curve of LPC(14:0) concentrations between acute phase DILD patients and DILD-tolerant patients.

Next, we compared plasma LPC concentrations between acute and recovery phase of DILD patients and DILD-tolerant patients (Fig. 2A). All eight LPCs were expressed at significantly lower levels in patients with DILD in the acute phase than in the recovery phase or in DILD-tolerant patients. ROC analysis was then performed to evaluate the discriminative performance of the individual LPCs between acute and recovery phase DILD patients (Fig. 2B and Supplementary information 6A) and between acute phase DILD patients and DILD-tolerant patients (Fig. 2C and Supplementary information 6B). The calculated AUC was highest for LPC(14:0) in both comparisons (0.813 for recovery phase; 0.811 for DILD-tolerant), with Yoden index cut-off values of 277.2 ng/mL (recovery phase) and 273.9 ng/mL (DILD-tolerant). Other LPCs scored AUCs of 0.737–0.783 for recovery phase patients and 0.738–0.763 for DILD-tolerant patients. The correlation between LPC(14:0) and the other LPCs in DILD patients was strong, with correlation coefficient (r) ranging from 0.65 to 0.86 (data not shown). This indicates that LPCs show similar trends in response to DILD, and that scoring models with multiple LPCs would not achieve better discrimination. Moreover, the calculated AUCs revealed that LPC(14:0) performed better as a biomarker for DILD than KL-6 and SP-D (Supplementary information 7), suggesting that LPC(14:0) could be a novel, high-performance biomarker for DILD.

### Association between LPC(14:0) and the clinical characteristics of patients with DILD

**Figure 3:**
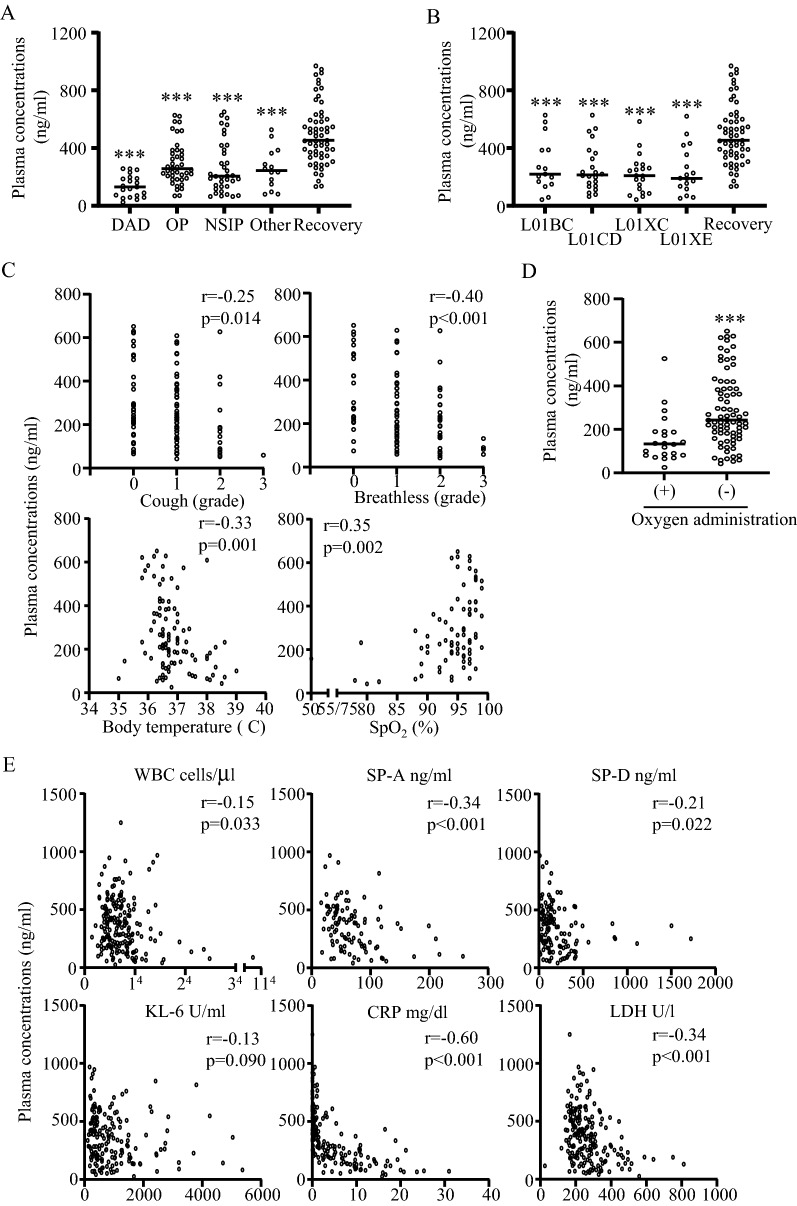
Association of LPC(14:0) with DILD patterns, causal drugs, symptom severities, and other DILD biomarkers. (**A**) Plasma LPC(14:0) concentrations for each DILD pattern in acute and recovery phase DILD patients. ***p < 0.001 *vs.* recovery phase. (**B**) Plasma LPC(14:0) concentrations in acute phase DILD patients treated with specific causal drugs and recovery phase DILD patients. L01BC: pyrimidine analogs; L01CD: taxanes; L01XC: monoclonal antibodies; L01XE: protein kinase inhibitors. ***p < 0.001 *vs.* recovery phase. (**C**) Plasma LPC(14:0) concentrations with cough and breathlessness scores, body temperatures, and SpO_2_ levels. (**D**) Plasma LPC(14:0) concentrations in acute phase DILD patients with or without oxygen administration. ***p < 0.001. (**E**) Plasma LPC(14:0) concentrations with other DILD biomarkers.

Plasma LPC(14:0) was next characterized in several aspects as a DILD marker. First, we investigated the relationship between DILD patterns and LPC(14:0) concentrations in acute and recovery of DILD patients. As shown in Fig. 3A, the DAD pattern had the strongest effect on plasma LPC(14:0) concentrations, but this effect was observed for the other DILD patterns. For the causal drug, the drug groups that belonged to the fourth level of the ATC code were selected to ensure statistical reliability, and the results with number of prescribed patients over 10 were shown (Fig. 3B). All groups of causal drug demonstrated a considerable impact on the plasma LPC(14:0) concentrations. A comparison of the acute phase and DILD-tolerant patients treated with the same group of causal drugs revealed that plasma LPC(14:0) concentrations were decreased due to DILD rather than the causal drugs (Supplementary information 8).

Furthermore, we examined the association between plasma LPC(14:0) concentration and severity of symptoms in DILD, by assessing parameters such as cough, breathlessness, body temperature, oxygen administration, and SpO_2_ levels. The severity of cough and breathlessness were evaluated by the attending physician based on the classification criteria for severity of adverse drug reactions^22^ and scored from 0 (no symptoms) to 3 (severe symptoms). SpO_2_ levels were only evaluated in patients without oxygen administration, since oxygen administration results in amelioration of SpO_2_ levels. As shown in Fig. 3C, all severity scores of DILD symptoms correlated significantly with plasma LPC(14:0) levels, with breathlessness levels exceeding the absolute correlation coefficient of > 0.4. In addition, patients with oxygen administration demonstrated significantly lower plasma LPC(14:0) concentrations than those without (Fig. 3D). These results suggest that plasma LPC(14:0) concentration is associated with those symptom severities in DILD that reflect disease severeity.

Along with DILD pattern, causal drugs, and symptom severities, we examined the association between plasma LPC(14:0) concentrations and other clinical chemistry/biomarker levels in patients with and without DILD. As shown in Fig. 3E, plasma LPC(14:0) concentration correlated significantly and negatively with blood C-reactive protein (CRP) levels (*r* = -0.60). Significant correlations were also observed with other biomarker levels, except KL-6, but with absolute correlation coefficients of < 0.4.

**Figure 4:**
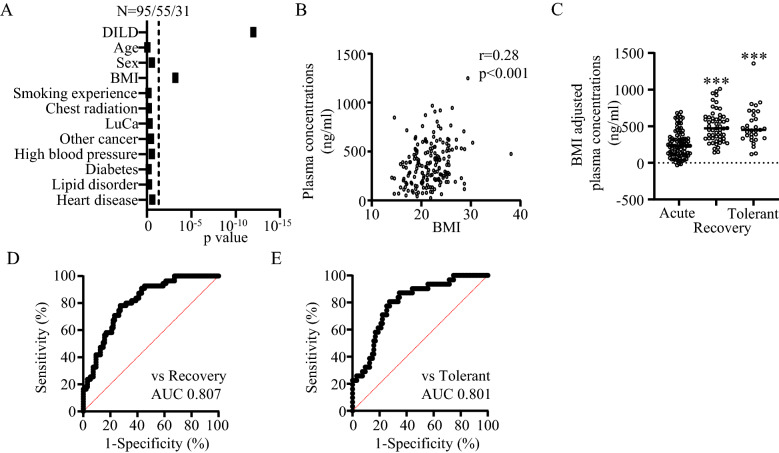
Association between LPC(14:0) and patient background characteristics. (**A**) Multivariable logistic regression analysis of the characteristic contribution to plasma LPC concentrations. (**B**) Plasma LPC(14:0) concentrations with BMI. (**C**) BMI-adjusted plasma LPC(14:0) concentrations in acute and recovery phase DILD and DILD-tolerant patients. ***p < 0.001 vs. acute phase. (**D**) ROC curve of BMI-adjusted LPC(14:0) concentrations between acute and recovery phase DILD patients. (**E**) ROC curve of BMI-adjusted LPC(14:0) between acute phase and DILD-tolerant patients.

Using multiple regression analysis, we examined the effects of the physical and clinical background characteristics of the patients, which may modulate the blood lipid profiles^23–25^. The physical characteristics examined were sex, age, body mass index (BMI), and smoking experience, and the clinical characteristics were chest radiation and associated diseases (LuCa, other cancers, high blood pressure, diabetes, lipid disorder, and heart disease). As shown in Fig. 4A, DILD was the primary factor contributing to plasma LPC(14:0) concentrations (*p* = 9.7E^-13^), and BMI was the only other factor that contributed significantly (*p* = 6.3E^-4^; Fig. 4B). To exclude the effect of BMI on plasma LPC(14:0) concentrations and evaluate the effect of DILD, we adjusted plasma LPC(14:0) concentrations for BMI using the beta obtained from multiple regression analysis and compared concentrations between acute and recovery phase DILD patients or DILD-tolerant patients. As shown in Fig. 4C, adjusted LPC(14:0) concentrations were significantly lower in patients with acute phase DILD than that in the recovery phase or DILD-tolerant patients. ROC analysis between acute and recovery phase DILD patients (Fig. 4D) and between acute phase DILD patients and DILD-tolerant patients (Fig. 4E) yielded AUCs of 0.807 for recovery phase patients and 0.801 for DILD-tolerant patients, consistent with unadjusted LPC(14:0) concentrations. These results suggest that BMI has a limited effect on plasma LPC(14:0) concentration and does not affect its application as a biomarker for DILD.

### Ability of LPC(14:0) to discriminate between DILD, other lung diseases, and healthy volunteers

**Figure 5:**
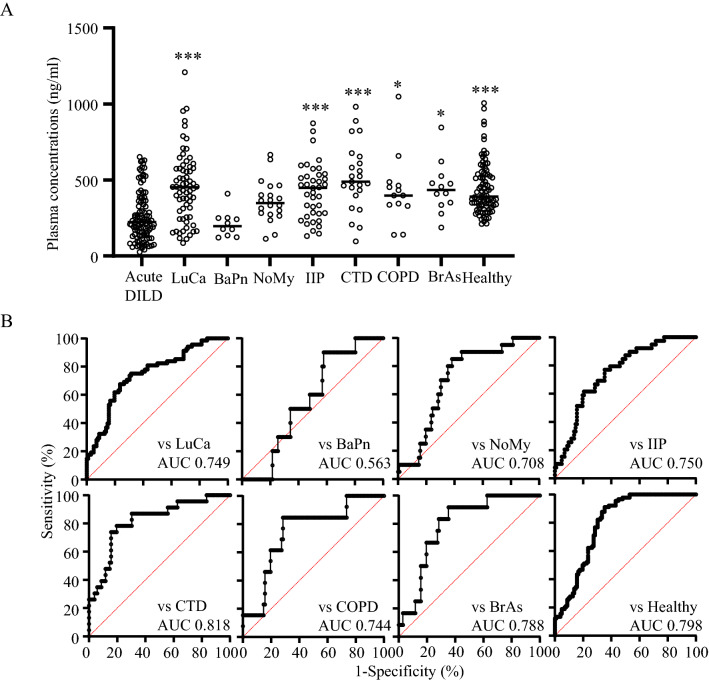
Ability of LPC(14:0) to discriminate DILD from other lung diseases and healthy volunteers. (**A**) Plasma LPC(14:0) concentrations in acute phase DILD patients, other lung diseases, and healthy volunteers. ***p < 0.001, *p < 0.05 *vs.* acute phase DILD. LuCa; lung cancer, BaPn; bacterial pneumonia, NoMy; nontuberculous mycobacteriosis, IIP; idiopathic interstitial pneumonia, CTD; lung disease associated with connective tissue disease, COPD; chronic obstructive pulmonary disease, BrAs; bronchial asthma, Healthy; healthy volunteer. (**B**) ROC curve of LPC (14:0) concentrations between acute phase DILD patients and other groups.

Finally, we examined the ability of plasma LPC(14:0) concentrations to discriminate between DILD and other lung diseases, LuCa (*n* = 68), BaPn (*n* = 10), NoMy (*n* = 20), IIP (*n* = 39), CTD (*n* = 23), COPD (*n* = 13), and BrAs (*n* = 12), or healthy volunteers (*n* = 90). Patient background characteristics are summarized in Table 2. Although there were several differences in background characteristics between patients with DILD and other lung diseases, multiple regression analyses demonstrated that background characteristics had a negligible effect (Fig. 4A). As shown in Fig. 5A, plasma LPC(14:0) concentrations were significantly lower in patients with acute phase DILD than in all other lung disease groups, except for BaPn and NoMy. ROC analysis (Fig. 5B) revealed that LPC(14:0) had substantial discriminative performance (> 0.7) between patients with acute phase DILD and other lung diseases, except for BaPn, and healthy volunteers (0.749 for LuCa, 0.563 for BaPn, 0.708 for NoMy, 0.750 for IIP, 0.818 for CTD, 0.744 for COPD, 0.788 for BrAs, and 0.798 for healthy volunteers). In addition, the AUCs against LuCa, IIP, and CTD revealed that LPC(14:0) performed better than KL-6 and SP-D (Supplementary information 9). The determined Yoden index cut-off values for LPC(14:0) were 368.1 ng/mL for LuCa, 274.3 ng/mL for IIP, and 392.6 ng/mL for CTD. These findings suggest that LPC(14:0) is an effective biomarker that can distinguish DILD from lung diseases, such as LuCa, IIP, and CTD, better than classical biomarkers.

**Table 2:** Summary of background information of patients with other lung diseases and healthy volunteers used for LPC biomarker assay.

Factor	Category	Lung cancer	Bacterial pneumonia	Nontuberculous mycobacteriosis	Idiopathic interstitial pneumonia	Lung disease associated with connective tissue disease	Chronic obstructive pulmonary disease	Bronchial asthma	Healthy volunteer
no. of subjects		68	10	20	39	23	13	12	90
Age	Range/median (no. of sub.)	44–81/70.5 (68)	55–81/73.5 (10)	48–83/64.5 (20)	41–83/72.5 (38)	50–83/68 (23)	51–80/66 (13)	42–87/60 (12)	25–65/47 (90)
Gender	Female/male (no. of sub.)	20/48 (68)	3/7 (10)	15/5 (20)	8/31 (39)	16/7 (23)	2/11 (13)	8/4 (12)	45/45 (90)
BMI	Range/median (no. of sub.)	15.9–30.4/21.9 (67)	16.5–29.3/22.8 (10)	12.7–24.7/18.7 (20)	15.7–32.4/24.3 (39)	19.9–44.3/23.7 (23)	13–29.4/21.1 (13)	18.7–32/24.5 (12)	18.4–25.6/21.4 (90)
Smoking habit	No/Yes (no. of sub.)	14/52 (66)	4/6 (10)	15/4 (19)	5/34 (39)	12/10 (22)	0/13 (13)	6/5 (11)	NA
Chest radiation	No/Yes (no. of sub.)	60/8 (68)	9/1 (10)	19/1 (20)	38/0 (38)	23/0 (23)	12/0(12)	12/0 (12)	NA

## Discussion

This study identified and characterized DILD biomarkers from patients with DILD, DILD-tolerant patients, patients with other lung diseases, and healthy volunteers. Lipidomics analysis screening and validation using patients with acute and recovery phase DILD revealed a systemic decrease in LPC class molecules, with larger effect size of saturated/monounsaturated fatty acid side chains in acute phase patients. Further analysis of plasma concentrations demonstrated that LPCs were present at significantly lower concentrations in the acute phase DILD, with LPC(14:0) exceeding the performance of the classical biomarkers KL-6 and SP-D. Notably, LPC(14:0) had no apparent association with causal drugs, or patient backgrounds and was associated with disease severity and CRP levels, unlike with KL-6 or SP-D levels. Furthermore, LPC(14:0) concentration can discriminate between patients with DILD and those with LuCa, IIP, CTD, COPD, and BrAs, exceeding the performance of KL-6 and SP-D biomarkers in LuCa, IIP, and CTD cases. Therefore, this study demonstrated that LPCs, particularly LPC(14:0), could be a novel and effective biomarker for DILD.

Blood LPC levels have previously been proposed as candidate biomarkers for acute inflammatory diseases. For instance, serum LPC levels are decreased in patients with community-acquired pneumonia and are associated with their prognosis and mortality^26,27^. Consistently, we found that plasma LPC(14:0) concentrations were decreased in patients with BaPn. LPCs have also been proposed as useful biomarkers for diagnosing sepsis and predicting patient mortality^28^, while metabolomics and lipidomics screening have recently revealed decreased serum LPC levels in patients with COVID-19 with lung injury^29,30^. Our results further strengthen the association between blood LPC levels and acute inflammation. Moreover, LPCs have been reported as candidate biomarkers for cancer and diabetes, with which chronic inflammation is associated^25,31,32^. However, we found that plasma LPC(14:0) concentrations were higher in patients with the chronic inflammatory lung diseases IIP, CTD, COPD, and BrAs, than that in patients with DILD. In addition, we demonstrated the negligible impact of cancers and diabetes on blood LPC(14:0) concentrations. Therefore, the association between chronic inflammation and blood LPC levels may be to a lesser extent than that of acute inflammation and blood LPC levels.

Notably, the estimated cut-off values of LPC(14:0) for discriminating between acute DILD and IIP were compatible with recovery phase DILD, or DILD tolerance, which was approximately 275 ng/mL. Consequently, LPC(14:0) could be useful for specifically diagnosing DILD compared to IIP, which have different treatment strategies. For LuCa and CTD, the estimated cut-off values of LPC(14:0) were around 380 ng/mL, which is over 100 ng/mL above the cut-off for DILD diagnosis. Whilst LPC(14:0) was not applicable for discriminating DILD from BaPn, culture-, antigen-, or gene-based tests for bacteria can be used for diagnosing BaPn.

To date, no direct mechanism-based evidence of decreased plasma LPC concentrations has been provided in patients with DILD. However, the roles of LPCs and their metabolizing enzyme autotaxin in inflammation and immune response have been well characterized^33,34^. Autotaxin acts as phospholipase D to cleave choline from LPC and produce lysophosphatidic acid (LPA), which activates various LPA receptors to induce inflammatory and anti-inflammatory responses^33,34^. Serum autotoxin levels have been reported to correlate with the severity and mortality of acute respiratory distress syndrome^35^, and increased serum autotoxin levels have been detected in patients with severe COVID-19^36^. Plasma autotaxin levels also correlate with mortality in patients with severe sepsis^37^, suggesting that autotaxin may play a role in systemic hyper-inflammation and decreased plasma LPC concentrations in DILD. However, autotaxin also exerts important effects in chronic inflammatory diseases, such as idiopathic pulmonary fibrosis (IPF)^34^, and is present at increased levels in the bronchoalveolar lavage fluid of IPF models^38^. Since our study demonstrated a lesser impact of chronic inflammation on plasma LPC levels than that of acute inflammation, the role of autotaxin on the plasma LPC levels during acute and chronic inflammation may be different and/or could involve other factors.

Along with the mechanism regulating the decreased in plasma LPC concentrations in patients with DILD, there is no direct evidence for the role of decreased LPCs themselves on DILD pathogenesis. However, LPC has been reported to activate multiple signaling pathways involved in oxidative stress and inflammatory responses^39^. The roles of LPCs in endothelial cells, including vascular endothelium, and immune cells were characterized in association with worsened inflammation. For example, LPCs induce the production of cytokines in monocyte recruitment, cytotoxicity, apoptosis, and oxidative stress in endothelial cells^40–43^ as well as those involved in macrophage and B cell activation, and apoptosis in immune cells^44–46^. Therefore, one possible role of reduced LPCs in DILD pathogenesis is the counteraction of lung inflammation.

Despite our novel findings, this study has several limitations. First, the sample sizes for patients with DILD and related lung diseases were relatively small for verified clinical assessment. Second, although we demonstrated that causal drugs had a limited impact on plasma LPC concentrations, we were unable to fully exclude the possibility that the primary effects of causal drugs and their associated diseases may have influenced our outcomes. Third, we recruited patients with DILD from four core hospitals using the same sampling protocol; however, hospital-to-hospital variation in sample preparation may have yielded slightly different plasma LPC concentrations. Fourth, we did not control the alcohol and food habits of the patients included in this study, which might have affected postprandial metabolite responses since alcohol intake and a high-fat diet have been shown to alter the expression of hepatic genes, including those related to energy homeostasis and diet metabolism^47,48^. Fifth, this study only included Japanese patients, and thus our findings may not be applicable to other ethnic groups. Finally, we did not examine the effects of different radiological patterns, such as the distributed area of the affected lung lesions, which may be associated with different inflammatory pathways as well as lipid alteration. Therefore, these limitations should be addressed in further studies before applying LPCs as practical biomarkers for diagnosing DILD in a clinical setting.

In conclusion, this study identified LPC(14:0) as a general biomarker of DILD, which was superior to KL-6 and SP-D when discriminating between acute and recovery phase DILD and tolerant controls. In addition, we characterized an association between LPC(14:0) and disease severity and demonstrated its ability to discriminate between DILD and IIP or CTD. Together, the findings of this study suggest that LPC(14:0) could help diagnose DILD before and during the administration of drugs indicated to treat the onset of DILD or in patients with suspected DILD having shortness of breath or dyspnea.

## Supplementary Information

Supplementary Information 1.

Supplementary Information 2.

Supplementary Information 3.

Supplementary Information 4.

Supplementary Information 5.

Supplementary Information 6.

Supplementary Information 7.

Supplementary Information 8.

Supplementary Information 9.

## Data Availability

All data generated or analysed during this study are included in this published article and its supplementary information files.

## Abbreviations

DILD: Drug-induced interstitial lung disease
LPC: Lysophosphatidylcholine
DAD: Diffuse alveolar damage
OP: Organizing pneumonia
NSIP: Non-specific interstitial pneumonia
CT: Computed tomography
IIP: Idiopathic interstitial pneumonia
HRCT: High-resolution CT
SP-D: Surface protein-D
KL-6: Krebs von den Lungen-6
LuCa: Lung cancer
CTD: Lung disease associated with connective tissue disease
BaPn: Bacterial pneumonia
NoMy: Nontuberculous mycobacteriosis
COPD: Chronic obstructive pulmonary disease
BrAs: Bronchial asthma
NIHS: National Institute of Health Science
FDR: False discovery rate
LC: Liquid chromatography
MS: Mass spectrometry
AUC: Area under the curve
ROC: Receiver operating characteristic
CRP: C-reactive protein
BMI: Body mass index
LPA: Lysophosphatidic acid
IPF: Idiopathic pulmonary fibrosis
