# Supplementary material for: Identification and characterization of lysophosphatidylcholine 14:0 as a biomarker for drug-induced lung disease
Source: Sci Rep. 2022 Nov 17;12:19819. doi: 10.1038/s41598-022-24406-z (PMC9671920; doi:10.1038/s41598-022-24406-z)
Supplement: Supplementary file 1 — Supplementary Information 1. [file 41598_2022_24406_MOESM1_ESM.docx]

**Supplementary information 1. Validation assay and its parameters and performance**

*Materials*

The following LPC standards, LPC(14:0), LPC(15:0), LPC(16:0), LPC(17:0), LPC(18:0), LPC(18:1), LPC(19:0), and LPC(20:0), and internal standard (IS) LPC(12:0), were purchased from Avanti Polar Lipids (Alabaster, AL, USA). Stock solutions were prepared at 500 μg/mL in methanol and stored at -80 °C until use. Bovine serum albumin (BSA) solution was purchased from Millipore Sigma (St. Louis, MO, USA) and phosphate-buffered saline (PBS) was purchased from Fujifilm Wako Pure Chemical Corporation (Tokyo, Japan). All other solvents and reagents were commercially available (LC/MS or HPLC grade).

*Preparation of LPC calibration standards*

Calibration standards were prepared by serial dilution with methanol at the following concentrations: 20, 40, 100, 200, 400, 1000, 2000, and 4000 ng/mL for LPC(14:0), LPC(15:0), and LPC(17:0); 1, 2, 5, 10, 20, 50, and 100 µg/mL for LPC(16:0); 0.4, 0.8, 2, 4, 8, 20, 40, and 80 µg/mL for LPC(18:0) and LPC(18:1); and 2, 4, 10, 20, 40, 100, 200, and 400 ng/mL for LPC(19:0) and LPC(20:0). All calibration standards were stored at -80 °C until use.

*Preparation of quality control (QC) samples*

Each pooled QC (QC1 to QC8) was prepared for each of the 51 patient samples, considering the availability of additional samples. Individual samples were ordered from high to low concentration of total LPC concentrations (QC1 to QC8). A lower limit of quantification (LLOQ) QC (QC9) was prepared by diluting pooled samples from 15 acute phase DILD patients 10× with 1% BSA in PBS. All QC samples were stored at -80 °C until use.

*Sample extraction*

LPCs were extracted from human plasma by protein precipitation using methanol. Human plasma was mixed with 99× the volume of methanol containing internal standard (IS) (2 ng/mL). An automated system (Microlab NIMBUS with MPE2 unit, Hamilton, Reno, NV, USA) was used to remove precipitated proteins by filtration with FastRemover for Protein (GL Science, Tokyo, Japan). The resulting filtrate was subjected to liquid chromatography/mass spectrometry (LC/MS).

*LC/MS*

Reverse-phase LC separation and multiple reaction monitoring (MRM) scans with quadrupole MS were used (LC; Ultimate 3000 system, MS; TSQ-Quantiva, Thermo Fisher Scientific, Waltham, MA, USA). An InertCore C18 column (2.4 μm, 2.1 × 50 mm; GL Science) was used for LC and temperature of the column oven was set at 50 °C. The temperature of the sample tray was maintained at 4 °C. Three microliters of sample was injected in each run. The solvent composition of the mobile phase was as follows: water with 10 mM ammonium formate (solvent A) and isopropanol with 10 mM ammonium formate (solvent B). The mobile phase was pumped at a flow rate of 0.3 mL/min with the following ramp gradient: 35% solvent B increased to 100% solvent B over 2.5 min, maintained at 100% mobile phase B for 0.9 min and equilibrated with 35% mobile phase B for 1.59 min before the next sample was injected. After separation, LPCs were subjected to MS in heated electrospray ionization mode with 3.5 kV spray voltage in positive ion mode with the following ion-source properties: sheath gas, 40 arbitrary units (Arbs); auxiliary gas, 10 Arbs; sweep gas, 1 Arb; ion-transfer tube temperature, 350 °C; and vaporizer temperature, 250 °C. MRM scan data were acquired using the following conditions: collision energy, 25 V; collision gas, Ar, 1.5; and transition, LPC(12:0) 440.28/184.058, LPC(14:0) 468.31/184.058, LPC(15:0) 482.32/184.058, LPC(16:0) 496.34/184.058, LPC(17:0) 510.36/184.058, LPC(18:0) 524.37/184.058, LPC(18:1) 522.36/184.058, LPC(19:0) 538.39/184.058, and LPC(20:0) 552.4/184.058 (m/z). Peak detection was performed with smoothing using TraceFinder 3.2 (Thermo Fisher Scientific, Waltham, MA, USA). After peak area quantification, a calibration curve was plotted using the standard data in GraphPad Prism 6 (GraphPad Software, San Diego, CA, USA).

*Validation*

We evaluated the following analytical parameters: calibration curve, carryover, parallelism, precision, relative accuracy, and stability of the QC samples.

Calibration curves were depicted using the mean normalized peak area (analyte/IS) of all calibration standards assayed in duplicate. A linear regression model with 1/X^2^ weighting was used as the equation of the calibration curve. The calibration curves were assessed using the relative error (RE, %) of the back-calculated concentration against the nominal concentration of each standard as follows: RE (%) = ((back-calculated concentration of the calibration standard sample − nominal concentration) / nominal concentration) × 100.

Carryover was assessed by comparing the peak area of the blank sample (methanol) injected just after the highest calibration standard concentration with that of the lowest calibration standard concentration, as follows: Ratio (%) of LPCs = (peak area of blank sample/peak area of LLOQ standard sample) × 100.

Parallelism was assessed from a single batch by calculating the slope ratio between the standard calibration curve and a response curve calculated using QC samples (Standards/QC samples).

The precision of the QC samples was assessed using the coefficient of variance (CV, %). Six replicates in one batch were used for intra-run analysis, whereas four individual batches were used for between-run analysis.

The accuracy of the QC samples (%) was assessed against the average of the determined concentrations in all batches. Intra-run accuracy (%) was assessed using six replicates in a batch, whereas between-run accuracy (%) was assessed using the mean concentrations of individual batches.

The stability of the QC samples was examined with respect to storage (long-term stability) and benchtop stability. Storage stability (%) was assessed by comparing the concentrations in samples stored for 12 months at -80 °C to those before storage. Benchtop stability (%) was assessed by comparing the concentrations in processed samples stored for 18 h at 4 °C to those in processed samples before storage.

Although fit-for-purpose acceptance criteria are crucial for biomarker assay validation and the validated performance of our assay was compatible with the criteria for drug approval by regulatory authorities (Reference S1-S3).

*Reference*

S1. US FDA. Guidance of industry: Bioanalytical method validation. 2018. Available from: http://www.fda.gov/files/drugs/published/Bioanalytical-Method-Validation-Guidance-for-Industry.pdf (Accessed on April 27th 2022)

S2. European Medicines Agency. Guideline on bioanalytical method validation. 2011. Available from: http://www.ema.europa.eu/en/documents/scientific-guideline/guideline-bioanalytical-method-validation_en.pdf (Accessed on April 27th 2022)

S3. Japan Ministry of Health, Labor and Welfare. Guideline on bioanalytical method validation in pharmaceutical development. 2013. Available from: http://www.pmda.go.jp/files/000206209.pdf (Accessed on April 27th 2022)

-6.
