# Supplementary material for: Identification and characterization of lysophosphatidylcholine 14:0 as a biomarker for drug-induced lung disease
Source: Sci Rep. 2022 Nov 17;12:19819. doi: 10.1038/s41598-022-24406-z (PMC9671920; doi:10.1038/s41598-022-24406-z)
Supplement: Supplementary file 6 — Supplementary Information 6. [file 41598_2022_24406_MOESM6_ESM.pptx]

## Slide 1
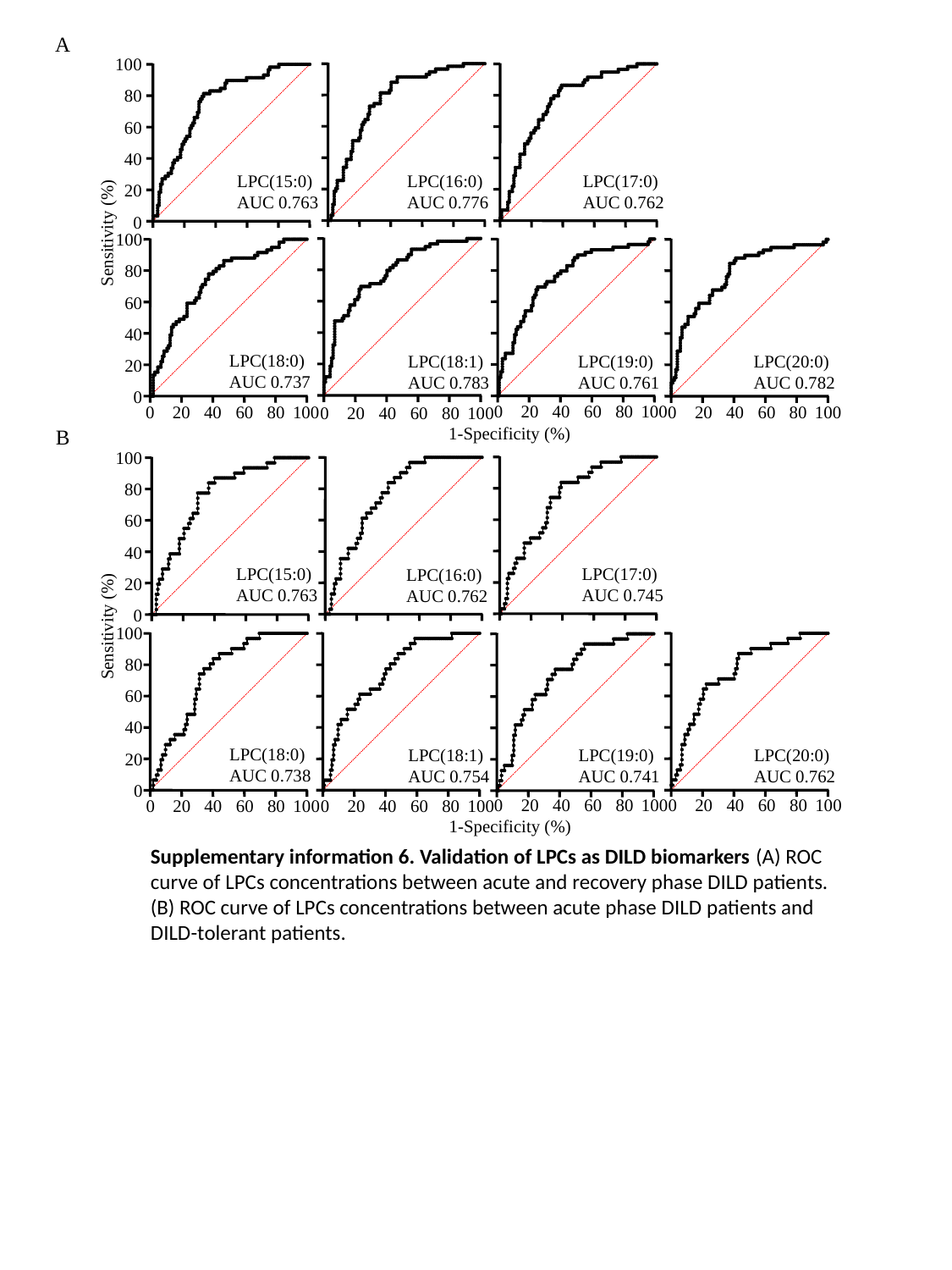

A
100
80
60
40
LPC(17:0)
AUC 0.762
LPC(15:0)
AUC 0.763
LPC(16:0)
AUC 0.776
20
0
Sensitivity (%)
100
80
60
40
LPC(18:0)
AUC 0.737
LPC(20:0)
AUC 0.782
LPC(18:1)
AUC 0.783
LPC(19:0)
AUC 0.761
20
0
0
20
40
60
80
100
0
20
40
60
80
100
0
20
40
60
80
100
0
20
40
60
80
100
B
1-Specificity (%)
100
80
60
40
LPC(17:0)
AUC 0.745
LPC(15:0)
AUC 0.763
LPC(16:0)
AUC 0.762
20
0
Sensitivity (%)
100
80
60
40
LPC(18:0)
AUC 0.738
LPC(20:0)
AUC 0.762
LPC(18:1)
AUC 0.754
LPC(19:0)
AUC 0.741
20
0
0
20
40
60
80
100
0
20
40
60
80
100
0
20
40
60
80
100
0
20
40
60
80
100
1-Specificity (%)
Supplementary information 6. Validation of LPCs as DILD biomarkers (A) ROC curve of LPCs concentrations between acute and recovery phase DILD patients. (B) ROC curve of LPCs concentrations between acute phase DILD patients and DILD-tolerant patients.
