# Supplementary material for: Identification and characterization of lysophosphatidylcholine 14:0 as a biomarker for drug-induced lung disease
Source: Sci Rep. 2022 Nov 17;12:19819. doi: 10.1038/s41598-022-24406-z (PMC9671920; doi:10.1038/s41598-022-24406-z)
Supplement: Supplementary file 7 — Supplementary Information 7. [file 41598_2022_24406_MOESM7_ESM.pptx]

## Slide 1
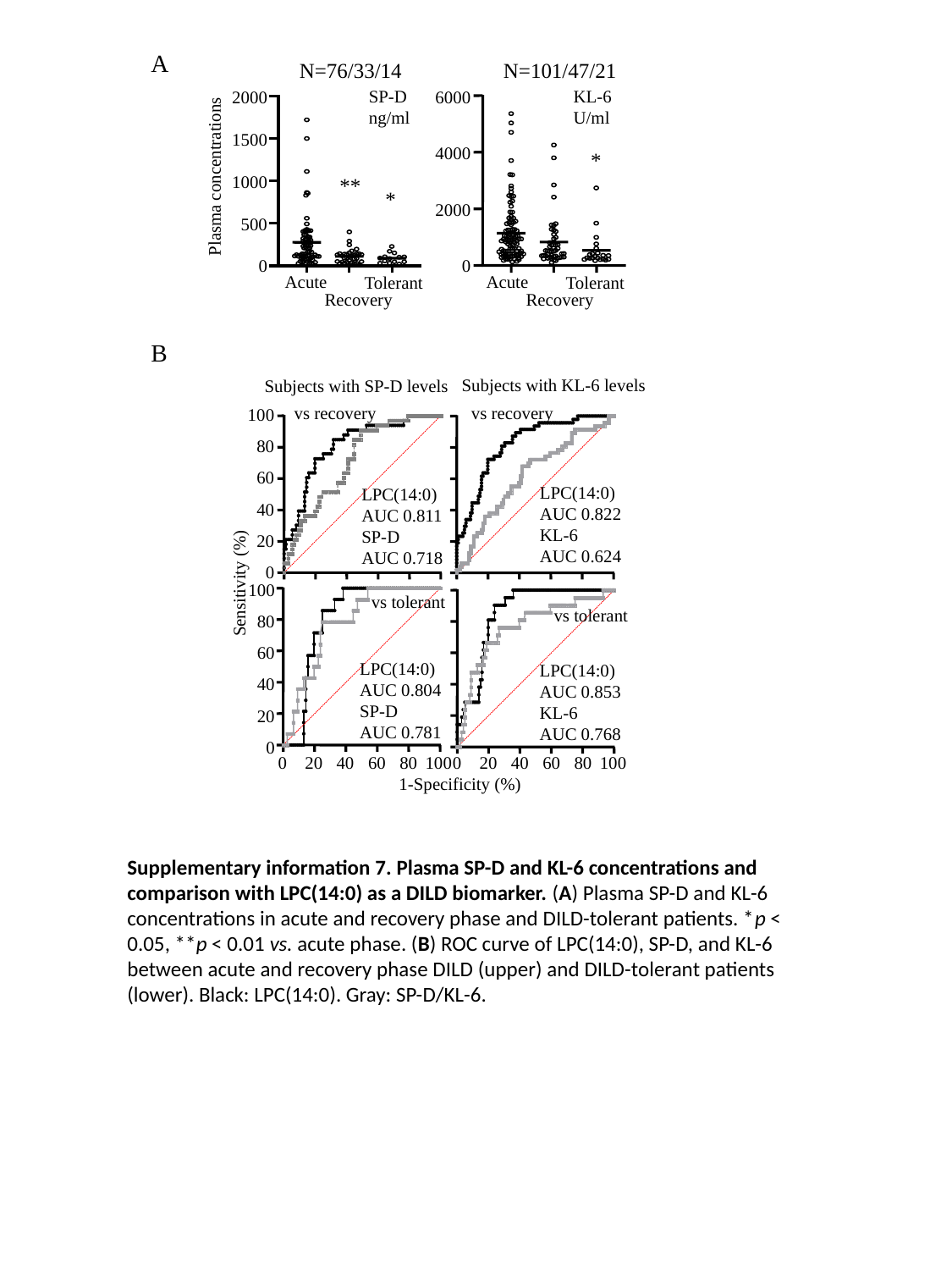

A
N=76/33/14
N=101/47/21
KL-6
U/ml
SP-D
ng/ml
6000
2000
1500
*
4000
Plasma concentrations
**
1000
*
2000
500
0
0
Acute
Acute
Tolerant
Tolerant
Recovery
Recovery
B
Subjects with KL-6 levels
Subjects with SP-D levels
vs recovery
vs recovery
100
80
60
LPC(14:0)
AUC 0.822
KL-6
AUC 0.624
LPC(14:0)
AUC 0.811
SP-D
AUC 0.718
40
20
0
Sensitivity (%)
100
vs tolerant
vs tolerant
80
60
LPC(14:0)
AUC 0.804
SP-D
AUC 0.781
LPC(14:0)
AUC 0.853
KL-6
AUC 0.768
40
20
0
0
20
40
60
80
100
0
20
40
60
80
100
1-Specificity (%)
Supplementary information 7. Plasma SP-D and KL-6 concentrations and comparison with LPC(14:0) as a DILD biomarker. (A) Plasma SP-D and KL-6 concentrations in acute and recovery phase and DILD-tolerant patients. *p < 0.05, **p < 0.01 vs. acute phase. (B) ROC curve of LPC(14:0), SP-D, and KL-6 between acute and recovery phase DILD (upper) and DILD-tolerant patients (lower). Black: LPC(14:0). Gray: SP-D/KL-6.
