# Supplementary material for: Identification and characterization of lysophosphatidylcholine 14:0 as a biomarker for drug-induced lung disease
Source: Sci Rep. 2022 Nov 17;12:19819. doi: 10.1038/s41598-022-24406-z (PMC9671920; doi:10.1038/s41598-022-24406-z)
Supplement: Supplementary file 8 — Supplementary Information 8. [file 41598_2022_24406_MOESM8_ESM.pptx]

## Slide 1
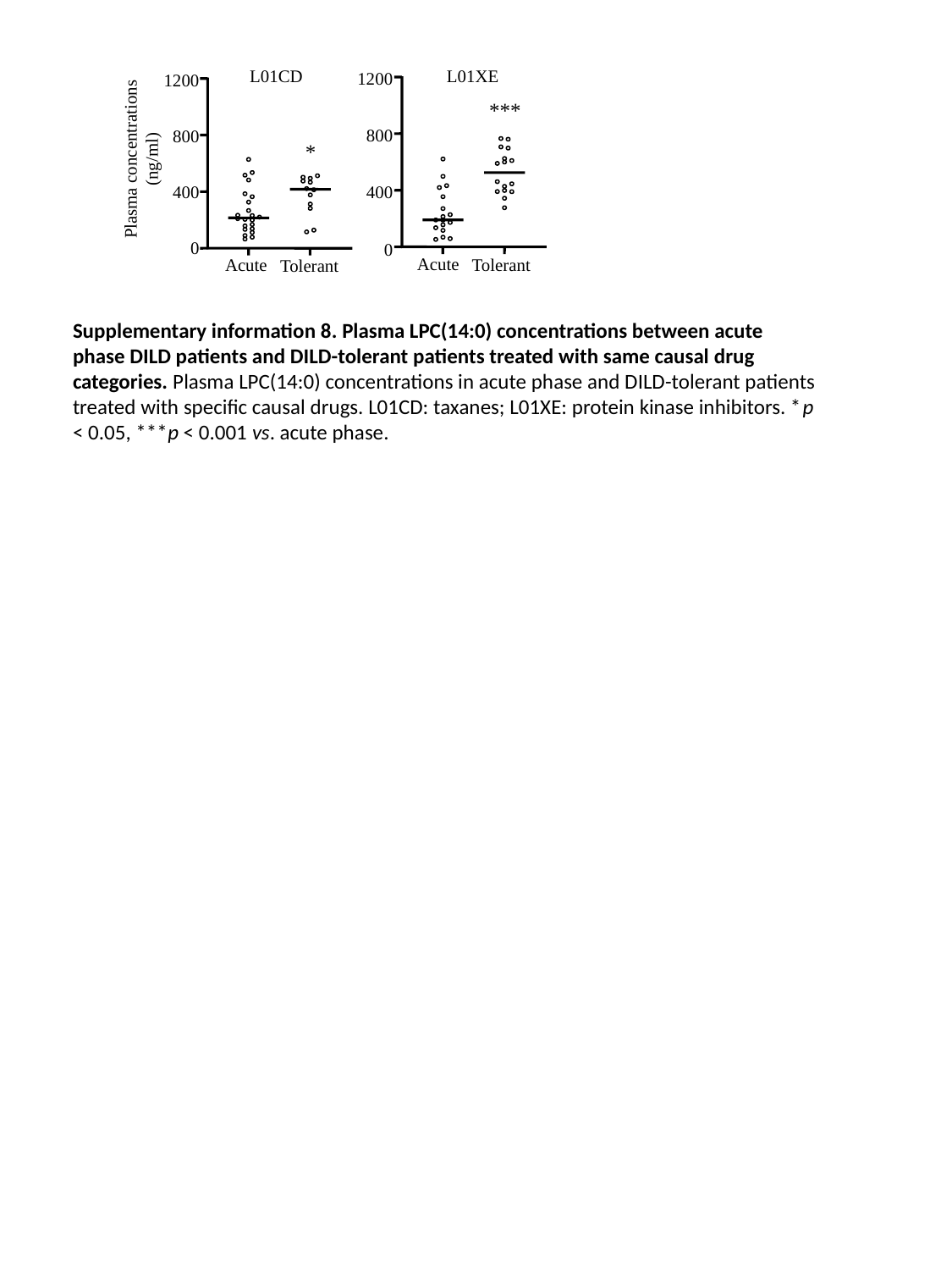

L01CD
L01XE
1200
1200
***
800
800
*
Plasma concentrations
(ng/ml)
400
400
0
0
Acute
Acute
Tolerant
Tolerant
Supplementary information 8. Plasma LPC(14:0) concentrations between acute phase DILD patients and DILD-tolerant patients treated with same causal drug categories. Plasma LPC(14:0) concentrations in acute phase and DILD-tolerant patients treated with specific causal drugs. L01CD: taxanes; L01XE: protein kinase inhibitors. *p < 0.05, ***p < 0.001 vs. acute phase.
