# Supplementary material for: Identification and characterization of lysophosphatidylcholine 14:0 as a biomarker for drug-induced lung disease
Source: Sci Rep. 2022 Nov 17;12:19819. doi: 10.1038/s41598-022-24406-z (PMC9671920; doi:10.1038/s41598-022-24406-z)
Supplement: Supplementary file 9 — Supplementary Information 9. [file 41598_2022_24406_MOESM9_ESM.pptx]

## Slide 1
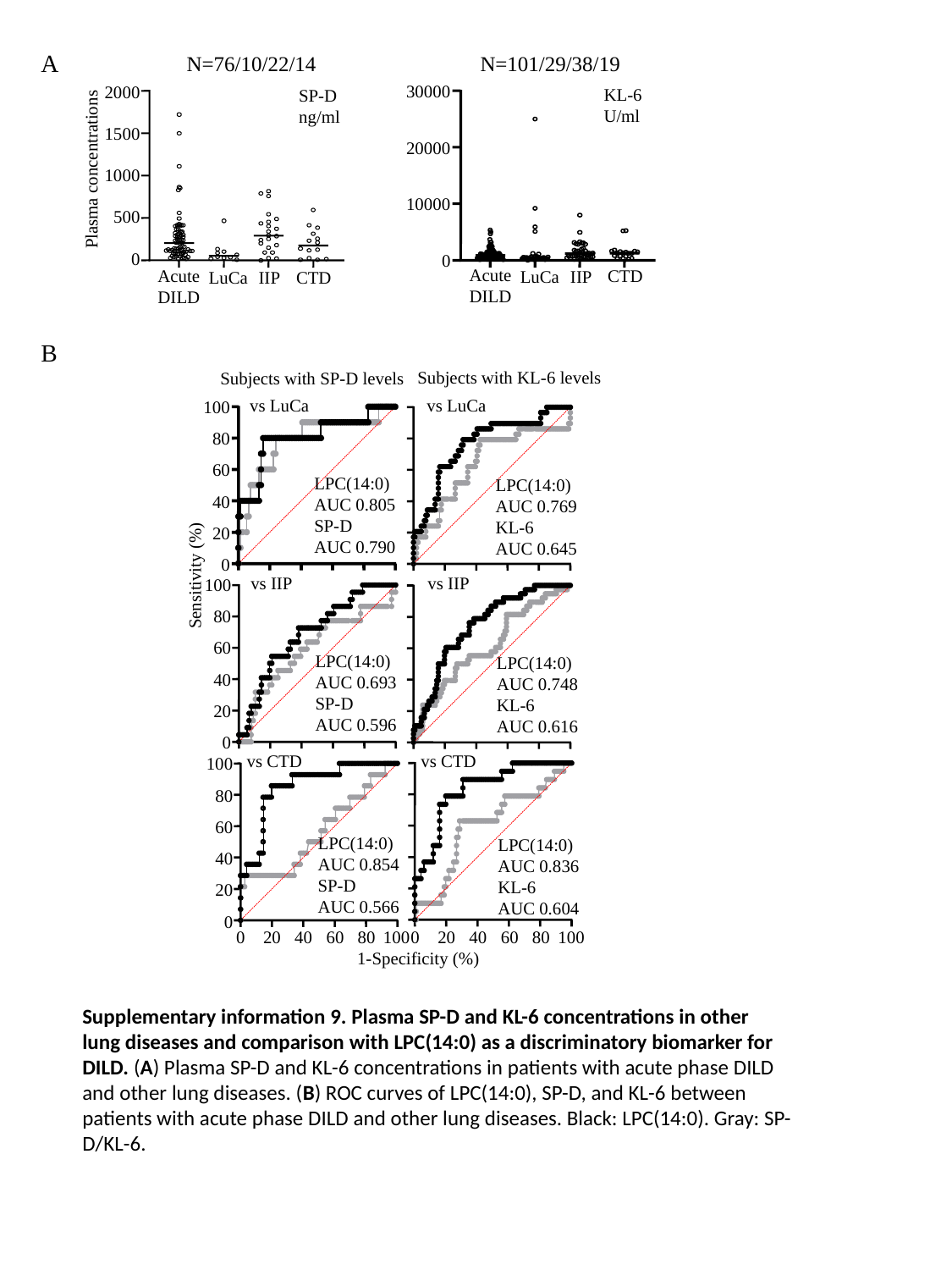

A
N=76/10/22/14
N=101/29/38/19
30000
2000
KL-6
U/ml
SP-D
ng/ml
1500
20000
Plasma concentrations
1000
10000
500
0
0
Acute
DILD
Acute
DILD
CTD
LuCa
IIP
CTD
LuCa
IIP
B
Subjects with KL-6 levels
Subjects with SP-D levels
vs LuCa
vs LuCa
100
80
60
LPC(14:0)
AUC 0.805
SP-D
AUC 0.790
LPC(14:0)
AUC 0.769
KL-6
AUC 0.645
40
20
0
Sensitivity (%)
vs IIP
vs IIP
100
80
60
LPC(14:0)
AUC 0.693
SP-D
AUC 0.596
LPC(14:0)
AUC 0.748
KL-6
AUC 0.616
40
20
0
vs CTD
vs CTD
100
80
60
LPC(14:0)
AUC 0.854
SP-D
AUC 0.566
LPC(14:0)
AUC 0.836
KL-6
AUC 0.604
40
20
0
0
20
40
60
80
100
0
20
40
60
80
100
1-Specificity (%)
Supplementary information 9. Plasma SP-D and KL-6 concentrations in other lung diseases and comparison with LPC(14:0) as a discriminatory biomarker for DILD. (A) Plasma SP-D and KL-6 concentrations in patients with acute phase DILD and other lung diseases. (B) ROC curves of LPC(14:0), SP-D, and KL-6 between patients with acute phase DILD and other lung diseases. Black: LPC(14:0). Gray: SP-D/KL-6.
